# Impact of a Three-Week ChatGPT 3.5-Generated Case-Based Professionalism Curriculum for Internal Medicine Residents at a United States Medical Residency: A Pilot Study

**DOI:** 10.7759/cureus.85401

**Published:** 2025-06-05

**Authors:** Jared J Bies, Sukhila Reddy, Monica Botros, Andrew C Stuart, Alok K Dwivedi, Brian P Edwards, Lisa A Hechanova, Debabrata Mukherjee, Abhizith Deoker

**Affiliations:** 1 Internal Medicine, Texas Tech University Health Sciences Center El Paso, El Paso, USA; 2 Paul L. Foster School of Medicine, Texas Tech University Health Sciences Center El Paso, El Paso, USA; 3 Molecular and Translational Medicine, Paul L. Foster School of Medicine, Texas Tech University Health Sciences Center El Paso, El Paso, USA; 4 Internal Medicine/Nephrology, Texas Tech University Health Sciences Center El Paso, El Paso, USA; 5 Internal Medicine, Paul L. Foster School of Medicine, Texas Tech University Health Sciences Center El Paso, El Paso, USA

**Keywords:** ai in medical education, case-based learning, chat gpt, professionalism assessment, professionalism in medical education

## Abstract

Objective: Artificial intelligence (AI), particularly language models like ChatGPT (OpenAI, San Francisco, CA), is reshaping clinical care and medical education. This study evaluated the impact of a ChatGPT 3.5-generated case-based curriculum on internal medicine residents’ understanding of professionalism in a US residency program.

Methods: A single-group, pre-post intervention pilot study was conducted from August 2024 to February 2025 at a US internal medicine residency program (IRB exempt, E24149). Residents from postgraduate year (PGY)-1 to PGY-3 participated in a three-week professionalism curriculum integrated into Friday ambulatory didactics. Weekly modules featured ChatGPT 3.5-generated case scenarios aligned with the Penn State Questionnaire on Professionalism (PSQP) domains, reviewed by three faculty members for clinical and ethical relevance. Residents completed one module per week via Qualtrics (Provo, UT), receiving immediate feedback. The validated 36-item PSQP was administered anonymously before and after the intervention. Pre-post differences were analyzed using unpaired t-tests adjusted for clustering based on baseline characteristics, with sensitivity analyses using log-transformed scores. Propensity score matching and cluster-adjusted logistic regression were used for subgroup analyses. Statistical significance was set at p < 0.05.

Results: A total of 37 residents completed the pre-survey, and 33 completed the post-survey. The mean age was 28.9 years (SD: 3.4), with balanced gender distribution (18 males, 19 females) and 59% non-US citizens. Residents were evenly distributed across PGY levels. After matching by age and sex, covariate balance was achieved. While all professionalism domains improved post intervention, changes were not statistically significant overall. Female residents showed significant gains in duty (p = 0.004), accountability (p = 0.037), honor (p = 0.028), and altruism (p = 0.017), with some effects persisting after matching. No significant changes were noted in male residents. Trends toward higher “much” or “great deal” responses were observed across most PSQP items (post: 61-77% vs. pre: 35-70%). Notable gains were seen in corrective action (p = 0.006), attending seminars (p = 0.003), and upholding scientific standards.

Conclusion: This pilot is among the first to evaluate a ChatGPT 3.5-generated professionalism curriculum using the validated PSQP. While overall changes were not statistically significant, meaningful gains in specific domains among female residents suggest educational benefit and support gender-responsive instructional design. The low-cost, scalable format may serve as a template for institutions seeking to implement professionalism training with limited resources. Further multi-institutional studies with paired designs and long-term follow-up are warranted.

## Introduction

Artificial intelligence (AI), particularly large language models (LLMs) like ChatGPT (OpenAI, San Francisco, CA), is rapidly transforming medical education. While AI’s diagnostic applications in clinical practice are well-established, its role in curriculum development, formative assessment, and learner engagement is an emerging and evolving area of research. Recent studies have demonstrated ChatGPT’s potential to generate board-style questions, simulate clinical case scenarios, and provide feedback that approximates expert-level reasoning, positioning it as a promising adjunct in medical training environments [1-3]. Educators are increasingly leveraging these tools to support personalized learning, streamline content development, and enhance reflective practice among trainees [4-6].

ChatGPT, developed by OpenAI, is part of the generative pre-trained transformer (GPT) family and was released as a public “research preview” on November 30, 2022. It quickly became one of the most widely accessible language models. Subsequent versions, including ChatGPT 3.5, expanded its capabilities and broadened its reach into both clinical and educational domains. Its integration raises important concerns about compliance with the Health Insurance Portability and Accountability Act (HIPAA), copyright protections, medico-legal accountability, and transparency in AI-generated content [7]. In clinical practice, ChatGPT has shown promise in decision support, improving clinical documentation, and optimizing workflow efficiency [5,8].

In education, ChatGPT has demonstrated utility in multiple domains. It can facilitate the generation of interactive learning modules, simulate virtual patient encounters, and assist in academic writing and curriculum design [5]. For instance, Eysenbach explored ChatGPT’s capabilities by prompting it to develop AI curricula, create quizzes, simulate patients, and propose academic integrity safeguards, underscoring its potential to innovate medical training [6]. Additionally, the model’s ability to perform well on United States Medical Licensing Examination (USMLE)-style questions highlights its aptitude for supporting standardized assessment preparation [1].

Professionalism, as one of the six core competencies defined by the Accreditation Council for Graduate Medical Education (ACGME), encompasses accountability to patients, society, and the profession; a commitment to ethical principles; and responsiveness to a diverse patient population [9]. Similarly, the American Board of Internal Medicine (ABIM) defines professionalism as the foundation of the physician-patient relationship and emphasizes placing patient interests above self-interest, maintaining competence and integrity, and fulfilling responsibilities to society [10].

Teaching professionalism has been incorporated into the curriculum of some residency programs. However, it remains particularly challenging to teach and assess due to its abstract, value-driven nature, which often lacks clearly defined behavioral benchmarks. Traditional methods, such as lectures or reflective essays, may be inconsistently applied and are difficult to standardize across programs. Furthermore, professionalism is context-sensitive, often demonstrated through subtle interpersonal behaviors and ethical decision-making that are not easily captured by conventional evaluation tools. These barriers are compounded in resource-limited settings where dedicated faculty time and curricular space may be scarce.

AI-based tools like ChatGPT offer a novel approach by enabling scalable, interactive, and case-based learning that simulates complex ethical dilemmas and professional scenarios. Such tools can be rapidly adapted to diverse training environments, provide real-time feedback, and promote active engagement, features that are particularly well-suited to teaching professionalism in a consistent yet personalized manner.

To our knowledge, there are currently no published studies that have systematically evaluated the use of AI-generated professionalism curricula within internal medicine residency programs. The aim of this pilot study was to explore the implementation of such an intervention using a ChatGPT 3.5-generated case-based curriculum and to examine its impact on residents’ understanding of professionalism using the validated Penn State Questionnaire on Professionalism (PSQP) tool [11]. Prior work has established the PSQP’s reliability and validity across medical learners, reinforcing its appropriateness for this investigation [12,13].

## Materials and methods

Formal IRB exemption was obtained under #E24149 prior to initiating research. Using AI’s freely accessible language model, ChatGPT 3.5, we administered an AI-generated three-week case-based professionalism curriculum. It was administered during residents’ ambulatory didactic sessions, a schedule chosen to align with the typical six-week rotation structure and to maximize participation across multiple cohorts. Although brief, the three-week duration was informed by previous studies that have successfully implemented short-format professionalism interventions and observed measurable changes in attitudes and awareness. The intervention was delivered to internal medicine residents across all three postgraduate years (PGY-1 to PGY-3), with representation from each level. Analyses were adjusted for PGY year and other baseline characteristics using propensity score matching.

This study utilized a single-group pre-post design without a control group, which limits the ability to draw causal inferences regarding the impact of the intervention. Changes observed in professionalism scores may have been influenced by external factors unrelated to the curriculum itself. While this design is appropriate for pilot studies aimed at assessing feasibility and generating hypotheses, future studies should incorporate randomized or controlled designs to strengthen internal validity and better isolate the effects of AI-driven educational interventions.

The three-week professionalism curriculum was integrated into the Friday morning didactic sessions during residents' ambulatory blocks between August 2024 and February 2025. Each week focused on different professionalism domains: week 1 emphasized altruism and accountability, week 2 focused on excellence and duty, and week 3 explored honor & integrity, and respect for others. Multiple-choice professionalism case scenarios were generated using ChatGPT 3.5 to create domain-specific vignettes grounded in the PSQP, a validated instrument comprising 36 items grouped into six key professionalism domains: altruism, accountability, duty, excellence, honor, and respect. For each domain, ChatGPT was prompted to generate clinical case scenarios based on the six grouped items associated with that domain. The goal was to simulate realistic situations that internal medicine residents might encounter and assess their responses through structured multiple-choice questions.

All scenarios generated by ChatGPT 3.5 were reviewed and refined by a panel of three internal medicine faculty members, including the program director and two associate program directors with experience in curriculum development and ethics education. Each reviewer independently assessed the scenarios for educational alignment with ACGME and ABIM core professionalism competencies, clinical appropriateness, ethical realism, and contextual relevance to internal medicine training. Discrepancies or suggested edits were discussed and resolved through group consensus. While formal inter-rater reliability (e.g., kappa statistics) was not calculated, content validation was achieved through iterative feedback and agreement among all three reviewers. Representative, faculty-approved scenarios from each professionalism domain are included in Appendices A-F, with one example presented in the main text.

A standard prompt used to generate each case was as follows: “Using the group item [insert group item] from the Penn State Questionnaire on Professionalism for the domain of [insert domain], create a clinical scenario for internal medicine residents. The scenario should include a brief vignette, five multiple-choice questions (one correct answer per question), explanations for each answer choice, and clear learning objectives linked to the PSQP domain.”

Twelve scenarios were generated for each domain and reviewed by faculty for clinical accuracy, ethical appropriateness, and educational alignment. Ten representative scenarios per domain were selected, and a complete example from the altruism domain is included below to meet transparency and replicability standards.

Representative case scenario - Altruism domain

Case Title: Financial Exploitation

Scenario: Dr. Carter, an internal medicine resident, is treating Mr. Smith, a long-term patient. Mr. Smith is deeply grateful for the care he has received and offers Dr. Carter a significant monetary gift to show his appreciation.

Question 1: Mr. Smith offers Dr. Carter a check for $5,000 as a token of his gratitude. What should Dr. Carter do first?

(a) Accept the check and thank Mr. Smith for his generosity.

Incorrect - Accepting large monetary gifts can lead to ethical concerns and conflicts of interest.

(b) Politely decline the check, explaining that accepting such gifts is against hospital policy and ethical guidelines.

Correct - This maintains professional boundaries and aligns with ethical standards.

(c) Suggest that Mr. Smith make a donation to the hospital instead.

Partially correct - Redirecting generosity helps, but it still involves a financial transaction.

(d) Accept the check but donate it to a charity without informing Mr. Smith.

Incorrect - Accepting the gift still violates policy, regardless of how it is used.

(e) Discuss the offer and suggest Mr. Smith use the funds for his own healthcare needs.

Partially correct - Shows concern for the patient, but does not address the boundary violation.

Learning objective: Understand the importance of maintaining professional boundaries and avoiding conflicts of interest by not accepting significant monetary gifts from patients.

Question 2: Mr. Smith insists on showing his appreciation financially. What should Dr. Carter suggest as an alternative?

(a) Accept a smaller gift, like a gift card, as a compromise.

Incorrect - Even small financial gifts may raise ethical concerns.

(b) Suggest Mr. Smith write a letter of appreciation to the hospital administration.

Correct - This provides a meaningful, non-financial way to express gratitude.

(c) Offer to reduce Mr. Smith’s medical bills as a favor.

Incorrect - Inappropriate and outside the resident's authority.

(d) Accept the check but promise to use it only for patient care expenses.

Incorrect - Still involves accepting the gift, which is against policy.

(e) Politely decline again and avoid suggesting any alternatives.

Partially correct - Maintains boundaries, but less constructive for patient engagement.

Learning objective: Promote appropriate ways for patients to express gratitude that do not involve financial gifts, thereby maintaining professional integrity.

Question 3: Mr. Smith decides to donate to the hospital in Dr. Carter’s name. How should Dr. Carter respond?

(a) Accept the donation in his name and ensure the hospital recognizes it.

Incorrect - Could still create a perceived conflict of interest.

(b) Thank Mr. Smith and ensure the donation is made anonymously or in honor of all healthcare workers.

Correct - Maintains professional boundaries and supports collective recognition.

(c) Refuse the donation altogether to avoid any issues.

Partially correct - Avoids conflict, but may seem dismissive of goodwill.

(d) Suggest Mr. Smith make a donation to a charity unrelated to the hospital.

Partially correct - Redirection helps, but feels impersonal.

(e) Accept the donation and suggest a small portion be used for hospital staff bonuses.

Incorrect - Inappropriate handling of donated funds.

Learning objective: Encourage ethical donations and ensure that they are made in a way that avoids any potential conflicts of interest or personal gain.

Question 4: Mr. Smith wants to continue expressing his gratitude and invites Dr. Carter to a private dinner. What should Dr. Carter do?

(a) Accept the dinner invitation to maintain a good patient-physician relationship.

Incorrect - Risks of blurring professional boundaries.

(b) Politely decline and suggest meeting in a professional setting if needed.

Correct - Maintains appropriate professional distance.

(c) Accept the invitation but bring a colleague along to keep it professional.

Partially correct - Involves oversight, but the setting may still be inappropriate.

(d) Decline the invitation and avoid all future contact.

Partially correct - Upholds boundaries, but may seem abrupt.

(e) Suggest a group lunch with hospital staff instead.

Partially correct - More professional, but still walks a fine line.

Learning objective: Maintain clear professional boundaries by avoiding private social interactions with patients that could lead to ethical issues.

Question 5: Dr. Carter reflects on the situation with Mr. Smith. What conclusion best demonstrates an understanding of professional boundaries?

(a) “I should have accepted the gift and the dinner to keep Mr. Smith happy.”

Incorrect - Demonstrates poor boundary awareness.

(b) “Maintaining professional boundaries is essential, even if it means refusing generous offers.”

Correct - Reflects ethical insight and professionalism.

(c) “It’s sometimes okay to accept gifts if they are given with good intentions.”

Partially correct - Intentions matter, but do not override policies.

(d) “I should have handled the situation differently to avoid awkwardness.”

Partially correct - Reflects concern for social dynamics, not ethics.

(e) “In the future, I’ll try to find a balance between professional boundaries and accepting gratitude.”

Incorrect - Suggests compromising on essential ethical principles.

Learning objective: Reflect on the importance of maintaining professional boundaries to ensure ethical and effective patient care.

Overall case objective: Understand the importance of maintaining professional boundaries to prevent conflicts of interest, ensure ethical patient care, and uphold the integrity of the medical profession.

Residents completed one professionalism module per week over a three-week period using the Qualtrics platform (Provo, UT). Each module was accessible for the duration of the assigned week, and residents were able to interact with the case content, answer multiple-choice questions, and receive immediate feedback. However, once submitted, responses could not be modified. Completion reminders were distributed weekly via email through program leadership. Module engagement was tracked using Qualtrics analytics, though specific completion percentages were not formally recorded.

The PSQP, a validated survey tool, was administered anonymously before and after the curriculum using the Qualtrics survey platform. PSQP includes a total of 36 items, and each item has five-point Likert responses (never, little, some, much, great deal). These 36 items measure professionalism in six domains, including accountability, altruism, duty, excellence, honor, and respect. Each of the six domains of PSQP ranges between 0 and 30 (higher is a better indicator of professionalism score). The scores were summarized with mean and standard deviation (SD) or median with interquartile range (IQR), while categorized scores were summarized with frequency and percentage. The post-intervention PSQP was administered immediately following the final case module during the third week’s scheduled Friday didactic session. This timing allowed for consistent exposure across cohorts but limited the analysis to immediate post-intervention attitudes.

Since the same residents were involved in pre- and post-surveys, we applied paired data analyses. Because of anonymity, we adopted data-driven approaches to form clustering/pairing of individuals based on their baseline characteristics. Because of the anonymous survey design, an unpaired t-test was used to compare pre- and post-intervention PSQP scores after accounting for clustering effects due to the pairing structure of the study design. Clusters are individuals paired between pre- and post-surveys using all baseline characteristics. In the primary analyses, we compared all untransformed outcome scores between groups. Considering the skewness in the scores, the pre- to post-comparisons were further evaluated on log-transformed scores using an unpaired t-test after accounting for clustering effects. The findings were validated by matching the pre-post data using the propensity score matching method. The propensity scores were developed for each resident using age and sex only by logistic regression analysis. The results of pre-post comparisons were summarized with the difference (d) in scores along with its 95% confidence interval and p-value. The score of individual items was further grouped into much or great deal versus the rest and compared between pre- and post-intervention groups using the cluster-adjusted logistic regression analysis. We also conducted sex specified analysis. Statistical analysis was conducted using Stata version 17 (StataCorp LLC, College Station, TX), and a p-value less than 0.05 was considered statistically significant.

## Results

Participant demographics

A total of 37 residents completed the pre-survey, and 33 completed the post-survey. The average age of the participants was 28.9 (SD: 3.4) years, with a similar distribution of gender ratio (18 males, 19 females). The participants had a slightly higher proportion of non-US citizenship (59%), with one-third of residents from each postgraduate year. After propensity matching based on age and sex, the distribution of covariates was not significantly different between pre- and post-surveys (Table 1).

**Table 1:** Baseline characteristics of residents between pre- and post-intervention after matching on age and sex. Moderate-size city (population: 50,000-500,000), rural (population: <2,500, not adjacent to a city), small city (population: 10,000-50,000), town (Population: 2,500-10,000), urban (population: > 500,000). B.S.: Bachelor of Science; D.O.: Doctor of Osteopathic Medicine; M.D.: Doctor of Medicine.

Factor	Pre-intervention	Post-intervention	p-value
N	32	32	
Age, mean (SD)	27.97 (2.13)	28.59 (1.86)	0.22
Credential			0.60
B.S.	1 (3%)	0 (0%)	
D.O.	5 (16%)	5 (16%)	
M.D.	26 (81%)	27 (84%)	
Postgraduate year			0.88
1	12 (38%)	11 (34%)	
2	12 (38%)	14 (44%)	
3	8 (25%)	7 (22%)	
Gender			0.31
Female	16 (50%)	13 (41%)	
Male	16 (50%)	17 (53%)	
Prefer not to say	0 (0%)	2 (6%)	
Hometown region			0.073
Moderate-size city	7 (22%)	2 (6%)	
Rural	4 (13%)	0 (0%)	
Small city	2 (6%)	2 (6%)	
Town	1 (3%)	2 (6%)	
Urban	18 (56%)	26 (81%)	
Citizenship			0.59
Non-United States	21 (66%)	23 (72%)	
United States	11 (34%)	9 (28%)	

Overall effect of the intervention

All professionalism domains showed improvement post intervention. However, the changes were not statistically significant in the overall group (Table 2).

**Table 2:** Overall differences in pre- and post-data using an unpaired t-test after adjusting for clustering effects.

	Pre-intervention (N = 31)	Post-intervention (N = 28)	p-value
	Mean (SD)	Mean (SD)	
Account	26.3 (3.9)	27.3 (3.6)	0.099
Altruism	26.9 (4.0)	27.6 (3.4)	0.240
Duty	26.1 (4.0)	27.1 (3.7)	0.174
Excellence	26.7 (3.8)	27.3 (3.8)	0.474
Honor	26.7 (3.8)	27.4 (3.7)	0.310
Respect	27.1 (3.6)	27.5 (3.7)	0.548

Subgroup analyses

Significant improvements were observed in female residents across several domains, including duty (25.8 vs. 28.2, d = 2.43; 95% CI: 1.05-3.80, p = 0.004), accountability (26.1 vs. 28.5, d = 2.39; 95% CI: 0.18-4.60, p = 0.037), honor (26.3 vs. 28.5, d = 2.29; 95% CI: 0.33-4.26, p = 0.028), and altruism (26.4 vs. 28.5, d = 2.11; 95% CI: 0.51-3.71, p = 0.017) (Table 3). Some of these remained statistically significant even after simply matching on age and sex using the propensity score method (Appendix G). In contrast, no significant improvement was noticed in male residents (Appendix H). These findings indicate a positive educational impact in specific learner subgroups.

**Table 3:** Differences in pre- and post-data among females using an unpaired t-test after adjusting for clustering effect. * P-values were calculated on log-transformed data.

	Pre-intervention (n = 16)	Post-intervention (N = 11)	p-value
Primary analysis	Mean (SD)	Mean (SD)	
Account	26.1 (4.0)	28.5 (2.3)	0.037
Altruism	26.4 (4.1)	28.5 (2.0)	0.017
Duty	25.8 (4.1)	28.2 (2.5)	0.004
Excellence	26.6 (3.9)	28.4 (2.4)	0.058
Honor	26.3 (4.1)	28.5 (2.1)	0.028
Respect	26.8 (3.9)	28.5 (2.3)	0.049
Validation analysis	Median (IQR)*	Median (IQR)*	
Account	26.5 (24.5, 29.5)	30.0 (26.0, 30.0)	0.027
Altruism	28.0 (24.0, 29.5)	30.0 (27.0, 30.0)	0.011
Duty	26.5 (23.0, 29.5)	29.0 (27.0, 30.0)	0.003
Excellence	28.0 (25.0, 30.0)	30.0 (27.0, 30.0)	0.044
Honor	28.0 (24.0, 30.0)	30.0 (27.0, 30.0)	0.019
Respect	28.0 (25.5, 30.0)	30.0 (27.0, 30.0)	0.037

Significant vs. non-significant items

**Figure 1:**
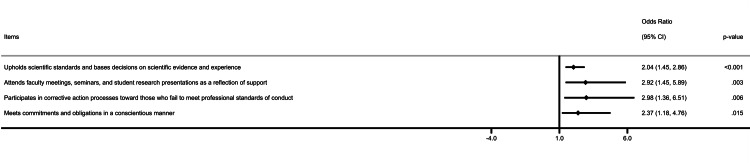
Most influential items following intervention in the overall cohort.

**Figure 2:**
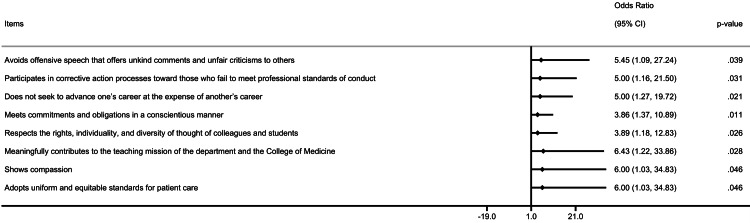
Most influential items following intervention in the female cohort.

The responses for most of the individual items were much or great deal, between 35% and 70% in the pre-intervention survey. In contrast, the responses for much or great deal were in the range of 61% to 77% following the ChatGPT 3.5 intervention. Across all domains of the PSQP, a trend toward increased endorsement of the "much" or "great deal" response options was observed for most individual items, although not all changes reached statistical significance. The significant and highest improvement was noticed for participation in corrective action (35% vs. 62%, p = 0.006), attending faculty meeting seminar (41% vs. 67%, p = 0.003), and upholding scientific standard (55% vs. 71%) in the entire cohort (Table 4). Similarly, multiple items of the PSQP were improved among females following the professionalism intervention through the AI approach (Appendix I). The most influential items following intervention in the overall cohort and the female cohort are shown in forest plots (Figures 1, 2).

**Table 4:** Overall differences in pre- and post-data (1: great deal vs. 0: not great deal).

Factor	Pre-intervention	Post-intervention	p-value
N	37	33	
Upholds scientific standards and bases decisions on scientific evidence and experience			<0.001
0	15 (45%)	9 (29%)	
1	18 (55%)	22 (71%)	
Maintains patient/physician relationships that do not exploit personal financial gain, privacy, or sexual advantages			0.115
0	10 (30%)	7 (23%)	
1	23 (70%)	24 (77%)	
Takes time to review other colleagues’ work and provides meaningful and constructive comments to improve it			0.371
0	17 (52%)	12 (39%)	
1	16 (48%)	19 (61%)	
Seeks self-improvement			0.144
0	12 (36%)	8 (26%)	
1	21 (64%)	23 (74%)	
Reports data consistently, accurately, and honestly			0.867
0	10 (30%)	9 (29%)	
1	23 (70%)	22 (71%)	
Avoids offensive speech that offers unkind comments and unfair criticisms to others			0.530
0	10 (30%)	8 (26%)	
1	23 (70%)	23 (74%)	
Shows a willingness to initiate and offer assistance toward a colleague’s professional and personal development			0.178
0	17 (53%)	10 (33%)	
1	15 (47%)	20 (67%)	
Promotes the welfare and development of junior faculty			0.555
0	14 (44%)	11 (37%)	
1	18 (56%)	19 (63%)	
Refusal to violate one’s personal and professional code of conduct			0.158
0	15 (47%)	7 (23%)	
1	17 (53%)	23 (77%)	
Appreciates and respects the diverse nature of research subjects and/or patients, and honors these differences in one’s work with them			0.079
0	16 (50%)	9 (30%)	
1	16 (50%)	21 (70%)	
Attends faculty meetings, seminars, and student research presentations as a reflection of support			0.003
0	19 (59%)	10 (33%)	
1	13 (41%)	20 (67%)	
Works collaboratively and respectfully within a team to the benefit of improved patient care or to the contribution of research			0.739
0	12 (38%)	10 (33%)	
1	20 (63%)	20 (67%)	
Participates in corrective action processes toward those who fail to meet professional standards of conduct			0.006
0	20 (65%)	11 (38%)	
1	11 (35%)	18 (62%)	
Does not seek to advance one’s career at the expense of another’s career			0.231
0	13 (42%)	9 (31%)	
1	18 (58%)	20 (69%)	
Volunteers one’s skills and expertise for the welfare of the community			0.215
0	16 (52%)	10 (34%)	
1	15 (48%)	19 (66%)	
Meets commitments and obligations in a conscientious manner			0.015
0	16 (52%)	9 (31%)	
1	15 (48%)	20 (69%)	
Respects the rights, individuality, and diversity of thought of colleagues and students			0.098
0	14 (45%)	8 (28%)	
1	17 (55%)	21 (72%)	
Meaningfully contributes to the teaching mission of the department and the college of medicine			0.475
0	15 (48%)	11 (38%)	
1	16 (52%)	18 (62%)	
Shows compassion			0.394
0	10 (32%)	6 (21%)	
1	21 (68%)	22 (79%)	
Demonstrates adaptability in responding to changing needs and priorities			0.378
0	12 (39%)	8 (29%)	
1	19 (61%)	20 (71%)	
Promotes justice in the healthcare delivery system by demonstrating efforts to eliminate discrimination in healthcare			0.796
0	12 (39%)	10 (36%)	
1	19 (61%)	18 (64%)	
Respects patient autonomy and helps them make informed decisions			0.834
0	8 (26%)	8 (29%)	
1	23 (74%)	20 (71%)	
Assumes leadership in patient management			0.203
0	12 (39%)	8 (29%)	
1	19 (61%)	20 (71%)	
Recognizes one’s own limitations			0.396
0	11 (35%)	8 (29%)	
1	20 (65%)	20 (71%)	
Assumes personal responsibility for decisions regarding patient care			0.458
0	12 (39%)	8 (29%)	
1	19 (61%)	20 (71%)	
Participates in activities aimed at attaining excellence in patient care			0.688
0	12 (39%)	10 (36%)	
1	19 (61%)	18 (64%)	
Reports medical or research errors			0.310
0	13 (42%)	9 (32%)	
1	18 (58%)	19 (68%)	
Acts in ways that show a commitment to confidentiality			0.959
0	9 (29%)	8 (29%)	
1	22 (71%)	20 (71%)	
Adopts uniform and equitable standards for patient care			0.622
0	12 (39%)	9 (32%)	
1	19 (61%)	19 (68%)	
Demonstrates empathy			0.957
0	9 (29%)	8 (29%)	
1	22 (71%)	20 (71%)	
Advocates a patient’s or research subject’s interest over one’s own interest			0.281
0	14 (45%)	9 (32%)	
1	17 (55%)	19 (68%)	
Discloses conflicts of interest in the course of professional duties and activities			0.410
0	12 (39%)	9 (32%)	
1	19 (61%)	19 (68%)	
Is professionally attired in a manner that is respectful of others			0.952
0	12 (39%)	11 (39%)	
1	19 (61%)	17 (61%)	
Responds to constructive criticism by working to improve one’s capability in the area criticized			0.731
0	11 (35%)	9 (32%)	
1	20 (65%)	19 (68%)	
Commits to implement cost-effective patient care			0.288
0	13 (42%)	9 (32%)	
1	18 (58%)	19 (68%)	
Represents information and actions in a truthful way			0.598
0	11 (35%)	9 (32%)	
1	20 (65%)	19 (68%)	

## Discussion

This pilot study evaluated the feasibility and short-term impact of a novel ChatGPT 3.5-generated professionalism curriculum in an internal medicine residency program using the validated PSQP. While pre-post differences in the overall cohort were not statistically significant, all six professionalism domains showed positive directional trends, suggesting potential educational benefit from AI-facilitated, case-based learning. Notably, female residents demonstrated statistically significant improvements in several domains, including duty, accountability, honor, and altruism, with large effect sizes. These findings remained significant even after propensity score matching for age and sex.

Item-level analysis further highlighted statistically significant improvements in behaviors such as participation in corrective actions, attending faculty seminars, and upholding scientific standards. These areas are often resistant to change through traditional didactics and suggest that interactive, scenario-based learning may promote deeper internalization of professional behaviors.

To contextualize these findings, we synthesized prior work using ChatGPT in medical education into four key domains: simulation, content generation, personalization, and formative assessment, each of which aligns with or contrasts meaningfully with our study.

Simulation and clinical reasoning

Prior studies have employed ChatGPT 3.0 and 3.5 for clinical case simulation in reasoning and high-acuity scenarios such as advanced cardiac life support (ACLS) and ICU care [2,14]. Our study builds on this by applying AI-generated simulations in the professionalism domain, which lacks standardized case-based teaching approaches. Unlike traditional simulation labs, AI-generated professionalism cases are cost-effective and scalable, providing exposure to ethically complex situations without logistical burden.

Content generation and curriculum development

ChatGPT’s use for content creation has been demonstrated in generating board-style questions, daily use during inpatient attending rounds, illness scripts, and expert discussion materials [3,15-17]. Our intervention highlights a novel extension: using ChatGPT to develop structured professionalism teaching modules aligned with a validated evaluation tool. Although faculty oversight remains essential to screen for inaccuracies and AI hallucinations, this method provides a scalable pathway for generating educational content in resource-limited settings [18,19].

Personalization and learner responsiveness

ChatGPT has been explored for its ability to deliver personalized educational experiences, particularly when guided by user-specific prompts [13]. Although our curriculum was standardized, the case-based format and embedded feedback encouraged individual reflection. The significant subgroup effects observed among female residents may indicate that such structured, reflective tools resonate more effectively with certain learner groups, an emerging area of interest for equity-driven medical education.

Formative assessment and feedback

The ability of AI to provide immediate, formative feedback supports learner engagement and self-assessment. In our intervention, residents received instant rationales tied to each case question, encouraging critical thinking and ethical reasoning in real time. This aligns with broader literature supporting the role of AI in facilitating formative learning loops that reinforce both knowledge and behavior change.

While ChatGPT offers new opportunities, it is also important to understand its place alongside existing professionalism education strategies. Traditional methods, such as didactic lectures and role modeling, have long been used to convey professional values, but they often lack consistency, structure, and scalability. Role modeling, while powerful, is highly variable and dependent on faculty availability and behavior, and didactics may not foster active engagement. In contrast, our AI-based curriculum provides a uniform, interactive, and scalable educational experience, promoting ethical reasoning through decision-making and immediate feedback. Thus, ChatGPT-based instruction serves as a complementary tool that enhances, not replaces, conventional professionalism teaching by filling gaps in standardization and learner autonomy.

While no randomized controlled trials (RCTs) are currently in progress concerning this topic, this study was structured as a pilot effort to assess feasibility, inform effect size estimates, and identify key variables for future controlled investigations. The insights gained from this intervention, including subgroup responsiveness, logistical feasibility, and domain-specific trends, can directly inform the design and implementation of a future RCT evaluating AI-generated professionalism curricula in graduate medical education [4].

This study is, to our knowledge, the first to evaluate a ChatGPT 3.5-generated professionalism curriculum using the validated PSQP tool [11]. Prior work has established the PSQP’s reliability and validity across medical learners, reinforcing its appropriateness for this investigation [12,13]. Our results extend the growing literature on AI-enhanced education by demonstrating the feasibility of using ChatGPT to address one of the most challenging and nuanced competencies in medical education - professionalism.

Limitations

This study has several potential limitations that must be considered. First, reliance on AI to generate professionalism case-based scenarios, though efficient, may result in content that lacks the depth, nuance, and contextual sensitivity of cases developed by experienced educators. The quality of AI-generated responses is dependent on prompt design and requires expert review to ensure accuracy, clinical appropriateness, and alignment with educational goals. To detect and address potential AI hallucinations, a known issue where language models generate plausible but incorrect information, all scenarios generated by ChatGPT 3.5 were reviewed and refined by a panel of three internal medicine faculty members, including the program director and two associate program directors with experience in curriculum development and ethics education [18,19]. Each reviewer independently assessed the cases for alignment with ACGME and ABIM core professionalism competencies, clinical and ethical realism, and contextual relevance to internal medicine training. Discrepancies or suggested edits were resolved through group consensus. While formal inter-rater reliability metrics (e.g., kappa statistics) were not calculated, content validation was achieved through iterative feedback and agreement among all reviewers. Legal and ethical considerations also remain relevant: while no protected health information (PHI) was used, the incorporation of AI tools in medical education raises concerns regarding HIPAA compliance, copyright infringement, and medico-legal accountability, particularly if such tools are integrated without oversight. Furthermore, the delivery of this curriculum via platforms such as ChatGPT and Qualtrics may limit scalability and reproducibility in institutions lacking similar technological infrastructure.

Beyond these AI-related concerns, there are several important methodological limitations. This was a single-group, pre-post study conducted at a single urban academic institution, which may limit external validity and generalizability to other training environments, such as rural programs or community-based institutions. The small, potentially non-representative sample further restricts the applicability of findings across diverse residency settings. The short duration of three weeks precluded any evaluation of long-term knowledge retention, behavioral change, or system-level outcomes. When framed within Kirkpatrick’s model of training evaluation, our study primarily targets level 2 (learning), capturing changes in knowledge and attitudes. It does not assess level 3 (behavior) or level 4 (results), such as sustained changes in professional conduct or patient care outcomes [20].

Additionally, the study’s non-randomized design does not control for confounding exposures during the intervention period. Residents may have been simultaneously exposed to other professionalism-related content, such as routine weekly didactic conferences, clinical feedback sessions with attendings, institutional graduate medical education (GME) professionalism town halls, or ACGME-mandated wellness and conduct modules, any of which could have influenced survey responses independently of the ChatGPT-based curriculum. These concurrent activities may have introduced unintended bias in the observed changes in attitudes.

Due to the anonymous nature of the surveys, individual pairing of pre- and post-responses was not possible, reducing the study’s statistical power and increasing potential response variability. Additionally, while statistical comparisons were made across multiple domains and subgroups, no correction for multiple testing was applied, which increases the likelihood of type I error. The female subgroup sample size (n = 13) was relatively small, limiting the reliability and generalizability of those statistically significant findings. Moreover, social desirability bias cannot be excluded, as residents may have been inclined to respond more favorably on professionalism-related items following exposure to the intervention.

Despite these constraints, the significant gains observed, particularly among female participants, suggest that even brief, AI-driven interventions may hold measurable educational value in teaching professionalism. These findings serve as preliminary, hypothesis-generating evidence that should be further evaluated in future multi-institutional RCTs. Incorporating qualitative feedback in future studies may also offer deeper insights into learners’ perceptions, experiences, and engagement with AI-generated professionalism content.

## Conclusions

To our knowledge, this is among the first studies to implement a ChatGPT 3.5-generated, case-based professionalism curriculum and evaluate its impact using a validated instrument, the PSQP, in a graduate medical education setting. While overall domain-level changes did not reach statistical significance, consistent upward trends and statistically significant improvements in specific domains among female residents suggest that AI-driven, case-based education may offer educationally meaningful benefits, particularly in structured and inclusive learning environments. This finding highlights the need for further exploration of how AI-based interventions may differentially impact learners and support gender-responsive educational design. The structured nature of the curriculum, low cost of content generation, and flexibility in delivery make this AI-based model highly scalable and adaptable, particularly for institutions seeking to implement or enhance professionalism training with limited faculty time or resources. This structured, AI-driven model may serve as a template for institutions aiming to deploy low-resource, standardized professionalism curricula. Future studies with larger, multi-institutional samples, paired pre-post survey designs, qualitative data, and long-term follow-up are needed to determine the effectiveness, sustainability, and broader implications of integrating large language models like ChatGPT into professionalism and competency-based medical education.

## Figures and Tables

**Figure 3:**
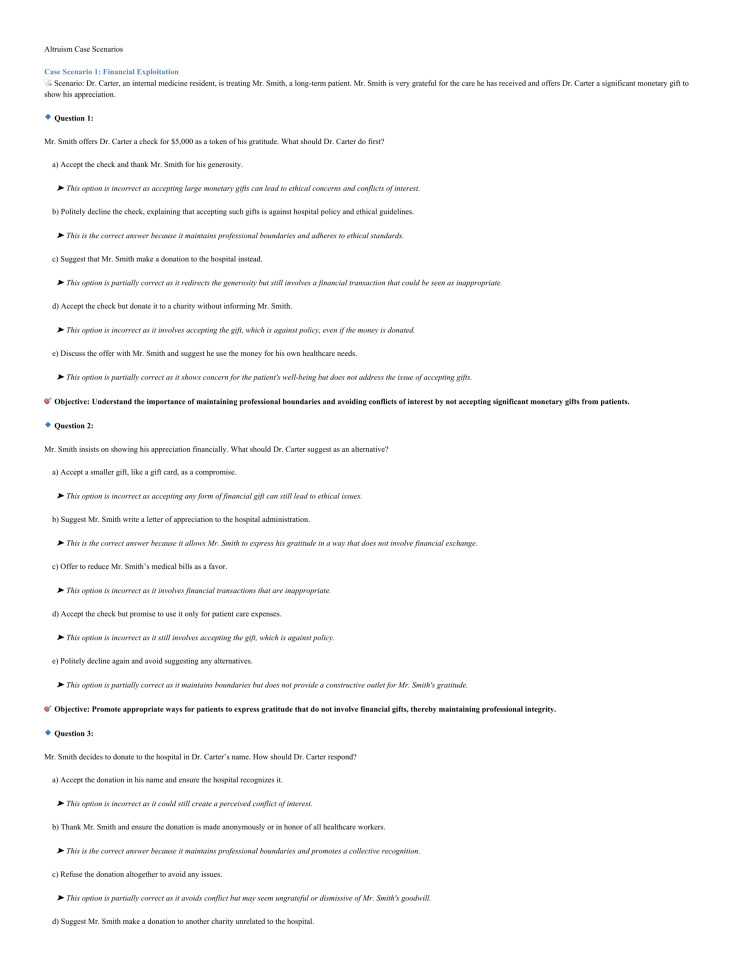
ChatGPT 3.5-generated professionalism curriculum – Altruism, case scenario 1: Financial exploitation part 1 of 2.

**Figure 4:**
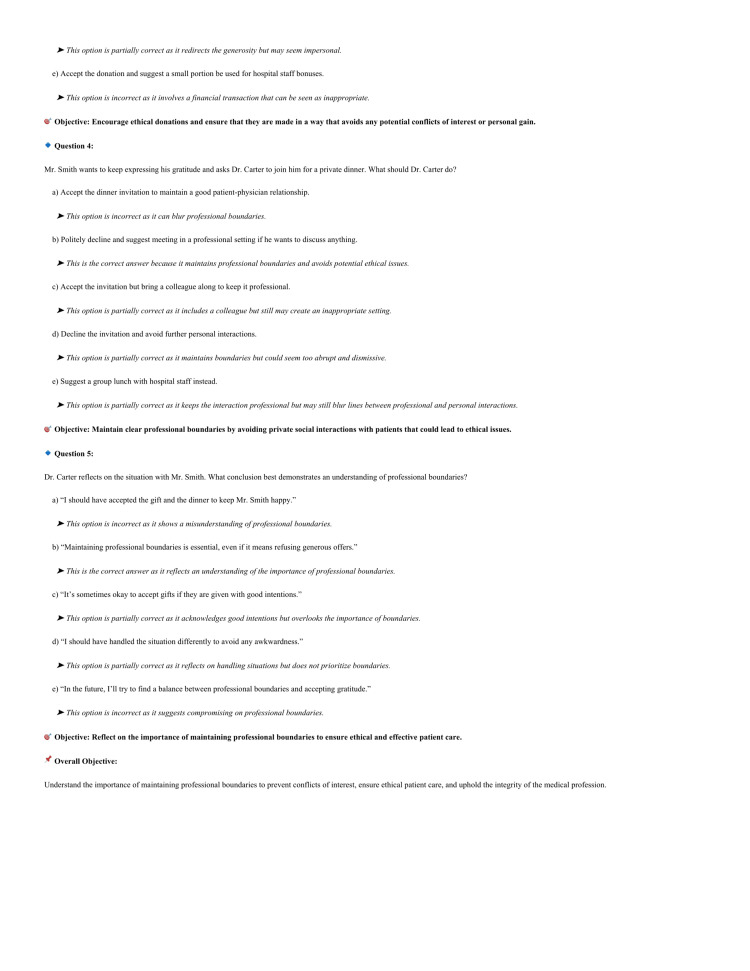
ChatGPT 3.5-generated professionalism curriculum – Altruism, case scenario 1: Financial exploitation part 2 of 2.

**Figure 5:**
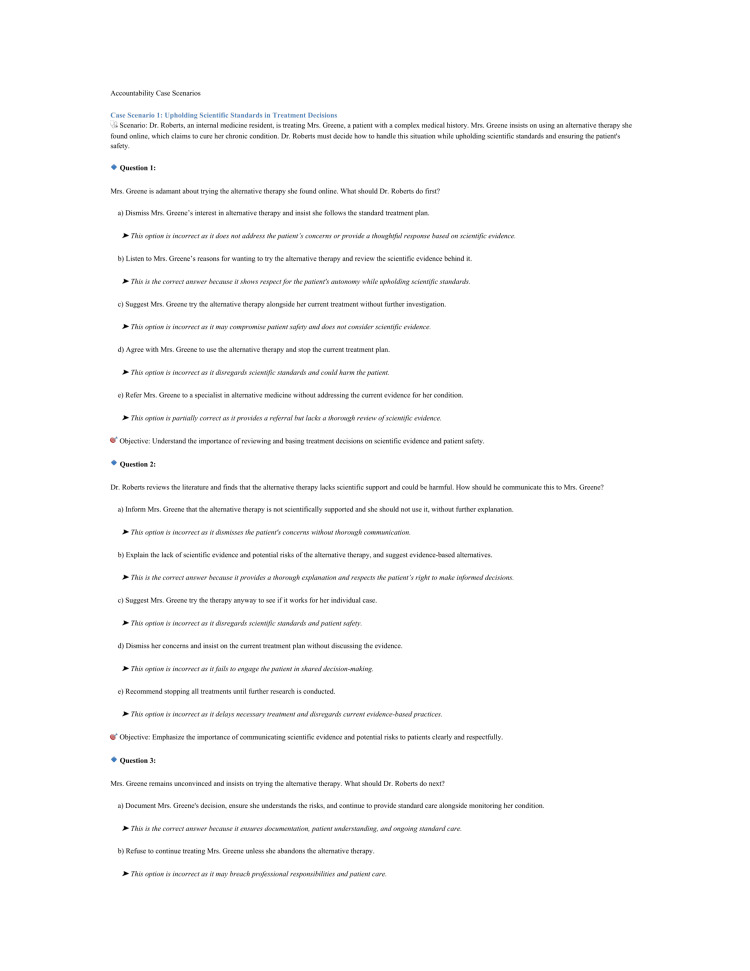
ChatGPT 3.5-generated professionalism curriculum – Accountability, case scenario 1: Upholding scientific standards in treatment decisions part 1 of 2.

**Figure 6:**
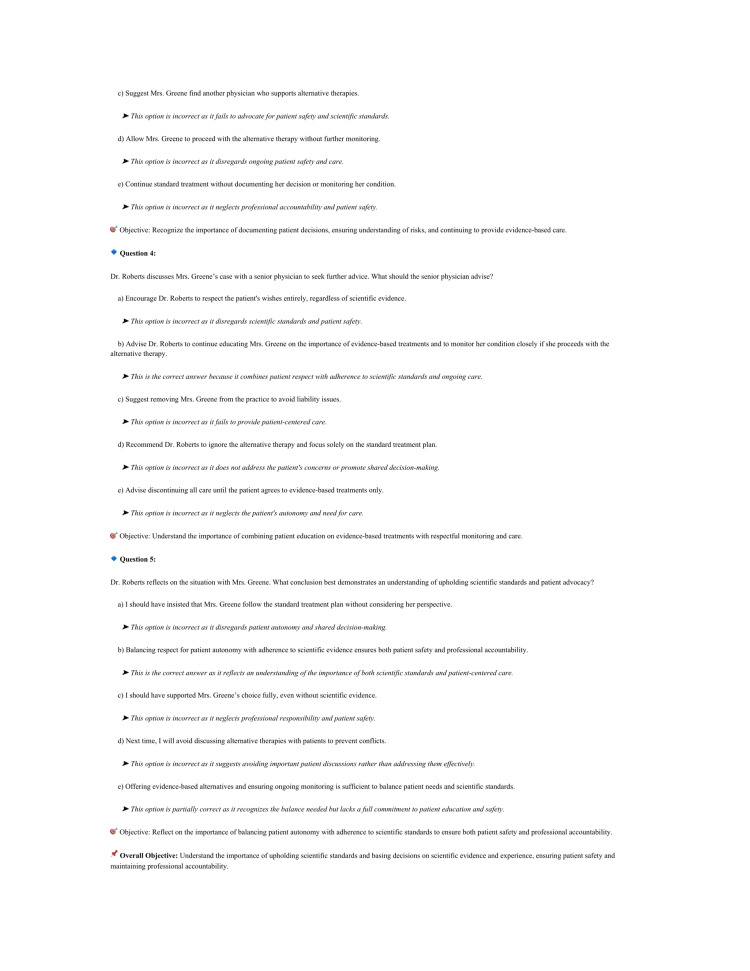
ChatGPT 3.5-generated professionalism curriculum – Accountability, case scenario 1: Upholding scientific standards in treatment decisions part 2 of 2.

**Figure 7:**
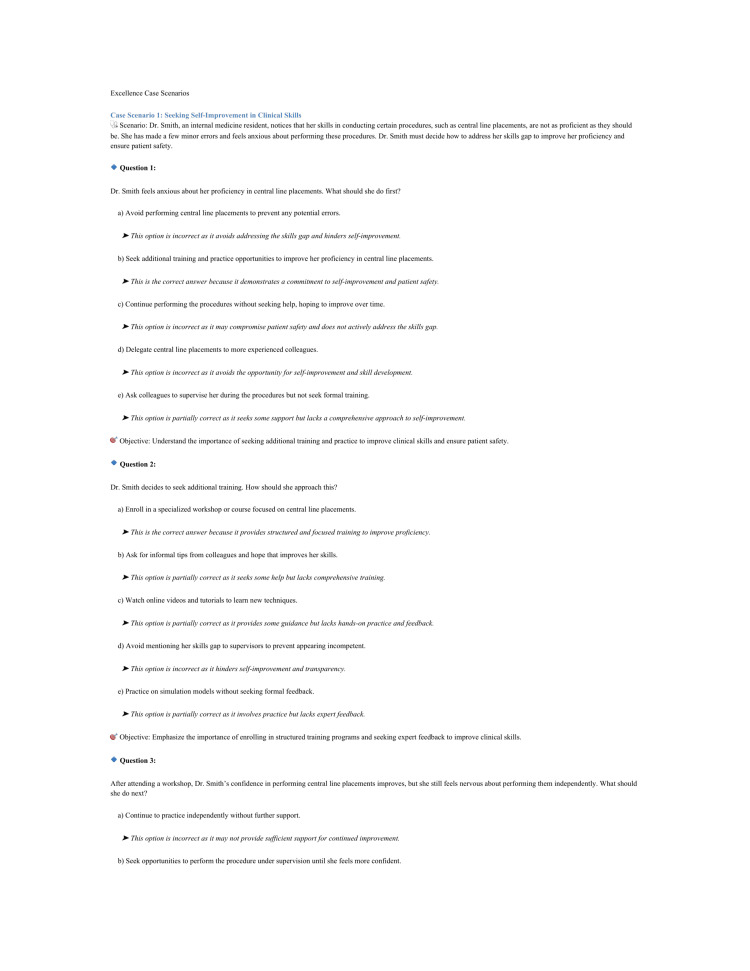
ChatGPT 3.5-generated professionalism curriculum – Excellence, case scenario 1: Seeking self-improvement in clinical skills part 1 of 2.

**Figure 8:**
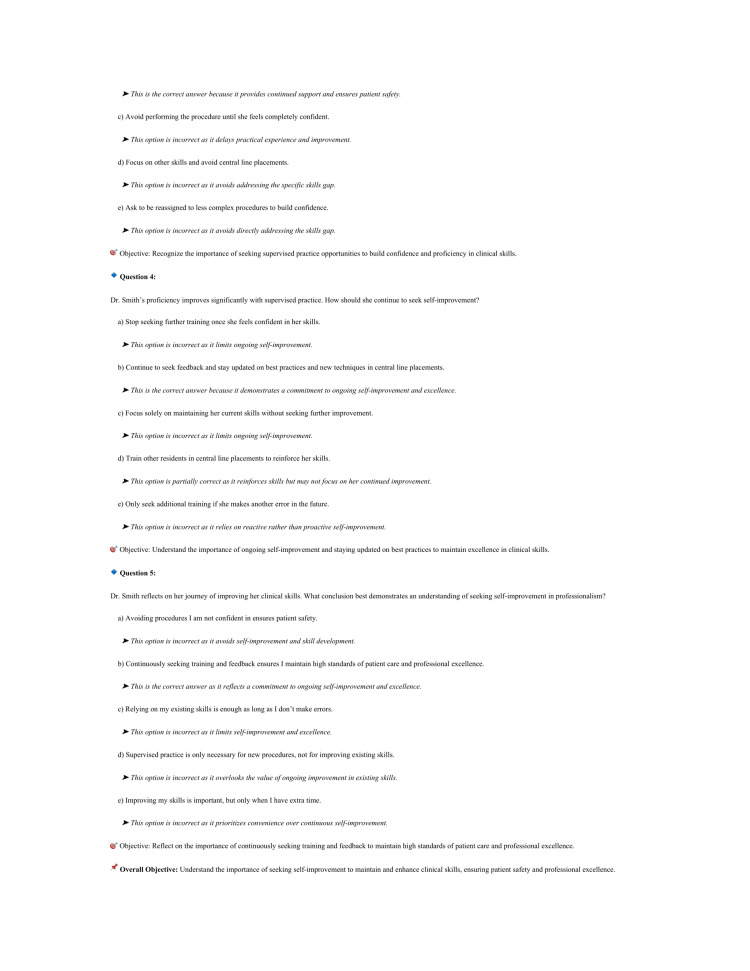
ChatGPT 3.5-generated professionalism curriculum – Excellence, case scenario 1: Seeking self-improvement in clinical skills part 2 of 2.

**Figure 9:**
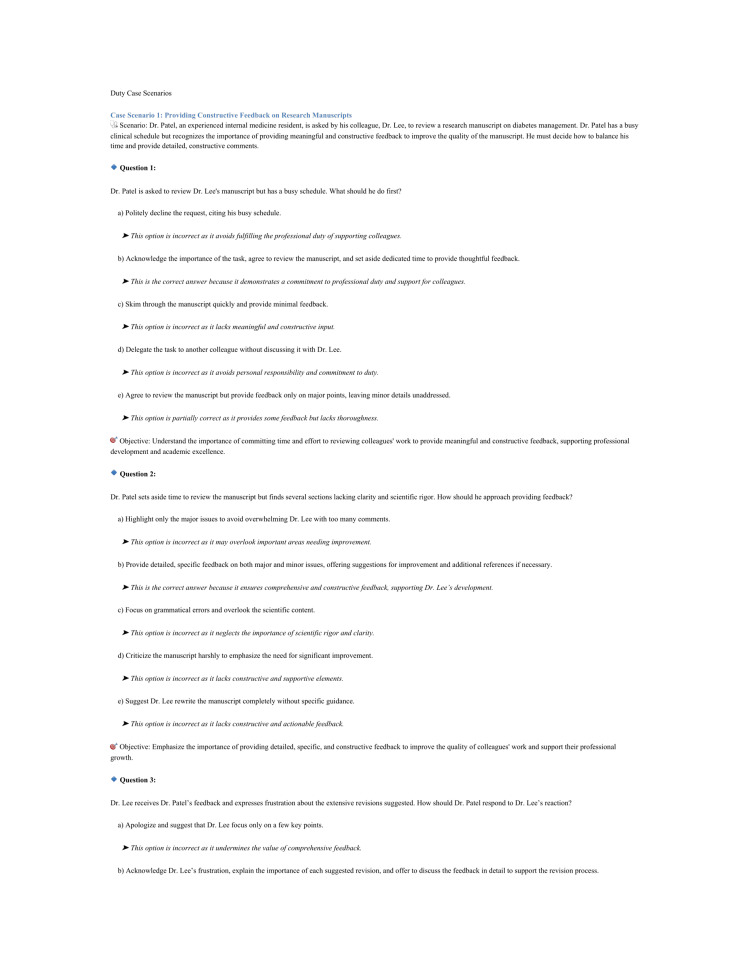
ChatGPT 3.5-generated professionalism curriculum – Duty, case scenario 1: Providing constructive feedback on research manuscripts part 1 of 2.

**Figure 10:**
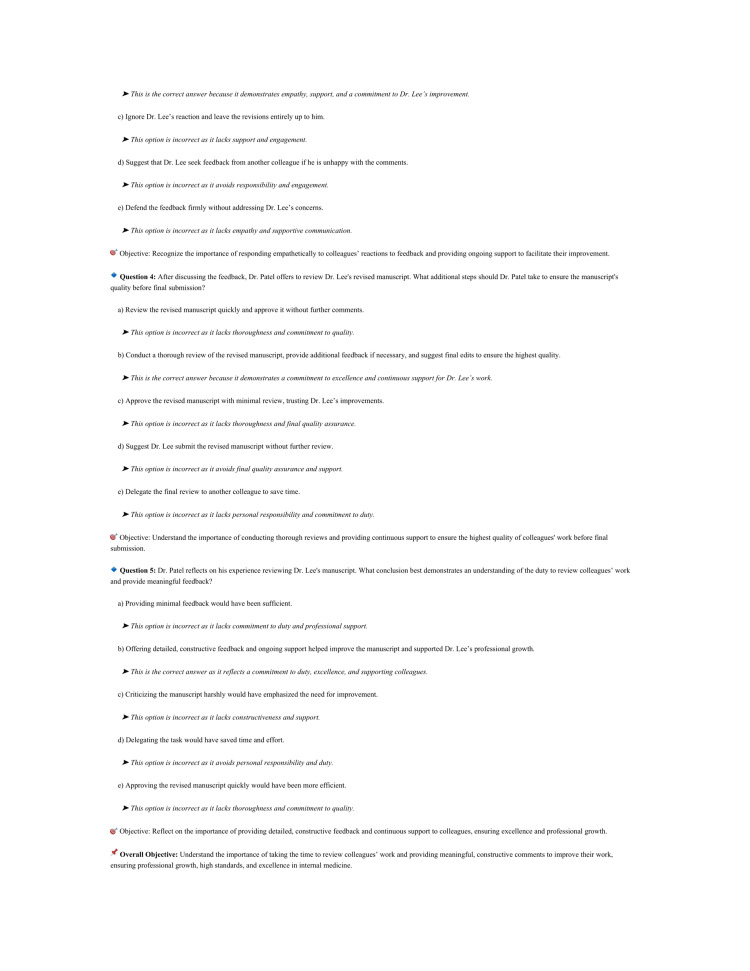
ChatGPT 3.5-generated professionalism curriculum – Duty, case scenario 1: Providing constructive feedback on research manuscripts part 2 of 2.

**Figure 11:**
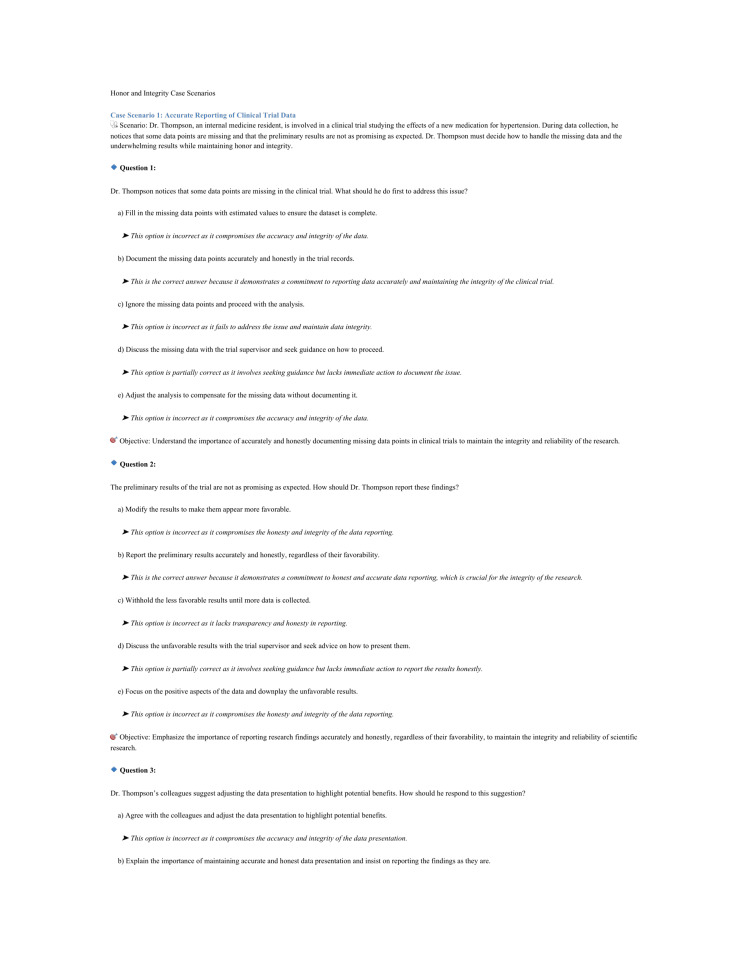
ChatGPT 3.5-generated professionalism curriculum – Honor & integrity, case scenario 1: Accurate reporting of clinical trial data part 1 of 2.

**Figure 12:**
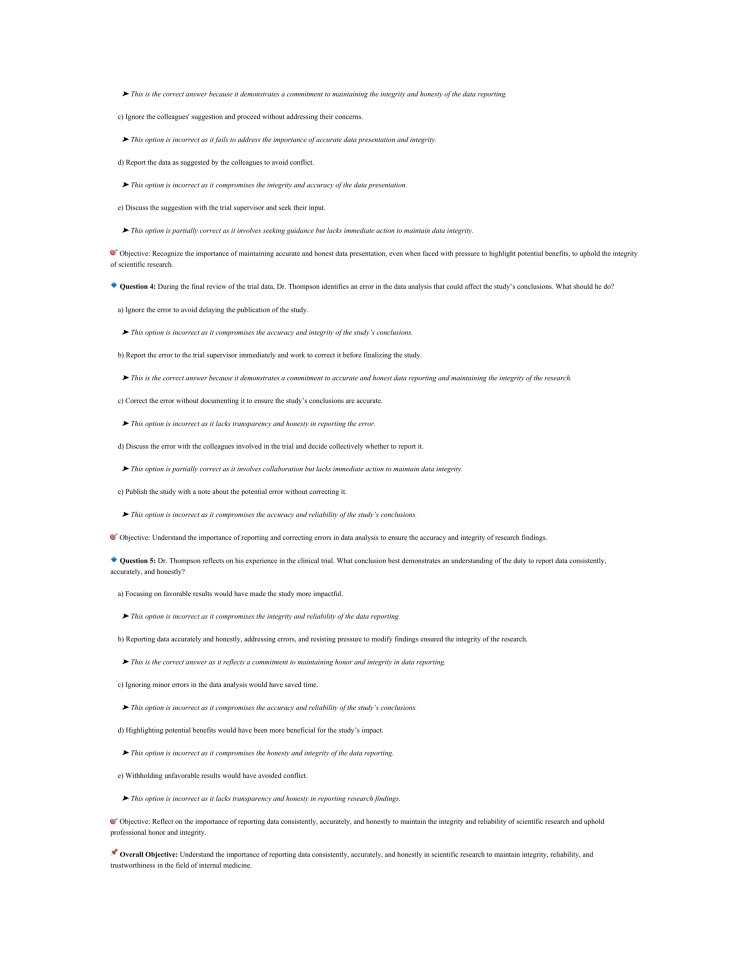
ChatGPT 3.5-generated professionalism curriculum – Honor & integrity, case scenario 1: Accurate reporting of clinical trial data part 2 of 2.

**Figure 13:**
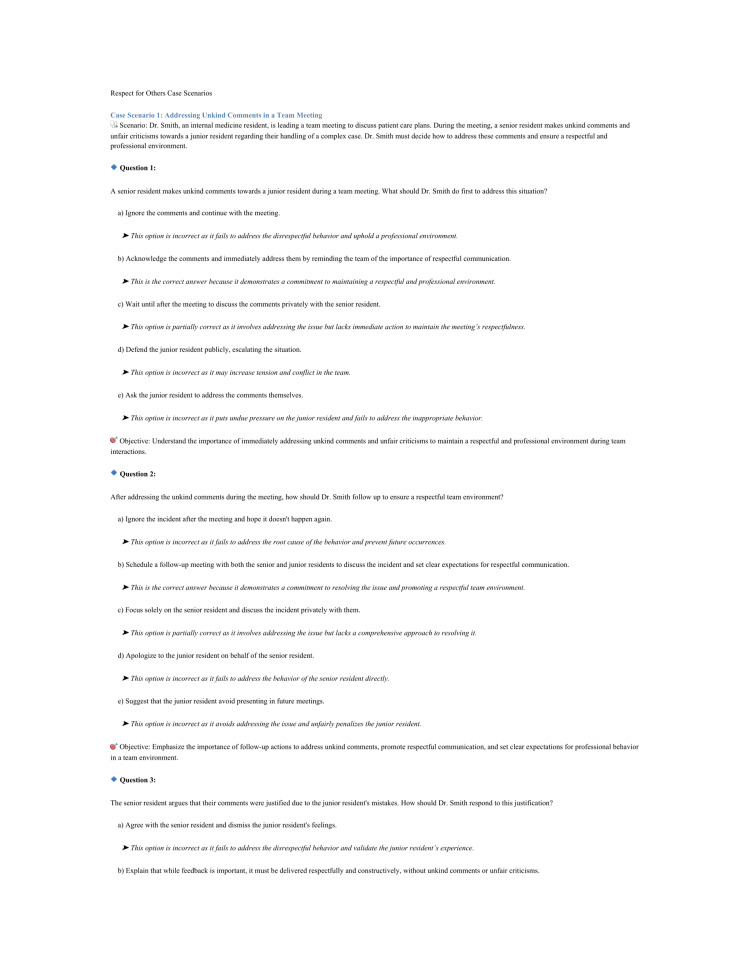
ChatGPT 3.5-generated professionalism curriculum – Respect for others, scenario 1: Addressing unkind comments in a team meeting part 1 of 2.

**Figure 14:**
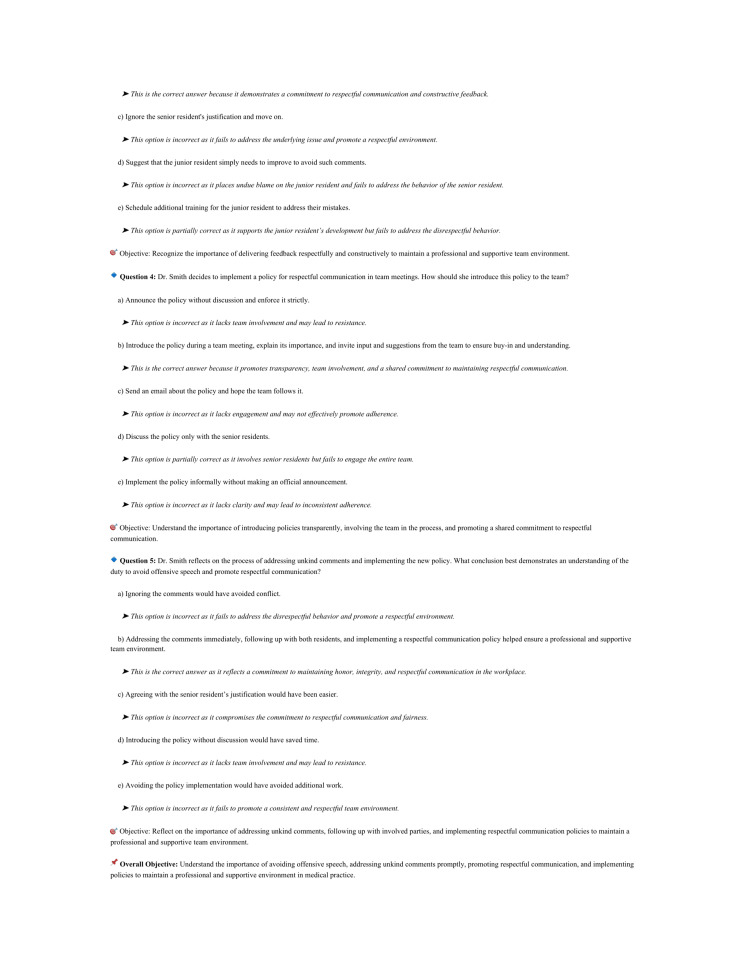
ChatGPT 3.5-generated professionalism curriculum – Respect for others, scenario 1: Addressing unkind comments in a team meeting part 2 of 2.

## Appendices

Appendix A

Appendix B

Appendix C

Appendix D

Appendix E

Appendix F

Appendix G

**Table 5:** Differences in pre- and post-data among females after matching on age and sex. * P-values were calculated on log-transformed data.

	Pre-intervention	Post-intervention	p-value
Primary analysis	Mean (SD)	Mean (SD)	
Account	25.8 (4.0)	28.5 (2.3)	0.042
Altruism	26.2 (4.1)	28.5 (2.0)	0.067
Duty	25.5 (4.1)	28.2 (2.5)	0.040
Excellence	26.3 (4.0)	28.4 (2.4)	0.100
Honor	26.0 (4.2)	28.5 (2.1)	0.051
Respect	26.5 (4.0)	28.5 (2.3)	0.108
Validation analysis	Median (IQR)*	Median (IQR)*	
Account	26.0 (24.0, 29.0)	30.0 (26.0, 30.0)	0.045
Altruism	28.0 (24.0, 29.0)	30.0 (27.0, 30.0)	0.066
Duty	26.0 (22.0, 29.0)	29.0 (27.0, 30.0)	0.041
Excellence	28.0 (25.0, 30.0)	30.0 (27.0, 30.0)	0.101
Honor	27.0 (24.0, 30.0)	30.0 (27.0, 30.0)	0.050
Respect	28.0 (25.0, 30.0)	30.0 (27.0, 30.0)	0.109

Appendix H

**Table 6:** Differences in pre- and post-data among males using an unpaired t-test after adjusting for clustering effects.

	Pre-intervention (N = 15)	Post-intervention (N = 15)	p-value
Primary analysis	Mean (SD)	Mean (SD)	
Account	26.5 (3.8)	26.9 (4.1)	0.615
Altruism	27.4 (3.9)	27.5 (3.9)	0.947
Duty	26.4 (4.0)	26.9 (4.3)	0.716
Excellence	26.9 (3.8)	27.0 (4.3)	0.951
Honor	27.2 (3.5)	27.3 (4.2)	0.906
Respect	27.5 (3.3)	27.3 (4.2)	0.854
Validation analysis	Median (IQR)*	Median (IQR)*	
Account	28.0 (24.0, 30.0)	29.0 (24.0, 30.0)	0.706
Altruism	30.0 (26.0, 30.0)	29.0 (26.0, 30.0)	0.954
Duty	27.0 (23.0, 30.0)	29.0 (25.0, 30.0)	0.76
Excellence	29.0 (24.0, 30.0)	30.0 (24.0, 30.0)	0.986
Honor	29.0 (24.0, 30.0)	30.0 (26.0, 30.0)	0.999
Respect	29.0 (25.0, 30.0)	30.0 (26.0, 30.0)	0.773

Appendix I

**Table 7:** Overall differences in pre- and post-data among females (1: great deal vs. 0: not great deal).

Factor	Pre-intervention	Post-intervention	p-value
N	18	13	
Upholds scientific standards and bases decisions on scientific evidence and experience			0.108
0	7 (44%)	3 (23%)	
1	9 (56%)	10 (77%)	
Maintains patient/physician relationships that do not exploit personal financial gain, privacy, or sexual advantages			0.465
0	4 (25%)	2 (15%)	
1	12 (75%)	11 (85%)	
Takes time to review other colleagues’ work and provides meaningful and constructive comments to improve it			0.251
0	8 (50%)	4 (31%)	
1	8 (50%)	9 (69%)	
Seeks self-improvement			0.298
0	5 (31%)	2 (15%)	
1	11 (69%)	11 (85%)	
Reports data consistently, accurately, and honestly			0.684
0	5 (31%)	3 (23%)	
1	11 (69%)	10 (77%)	
Avoids offensive speech that offers unkind comments and unfair criticisms to others			0.039
0	5 (31%)	1 (8%)	
1	11 (69%)	12 (92%)	
Shows a willingness to initiate and offer assistance toward a colleague’s professional and personal development			0.260
0	9 (56%)	4 (33%)	
1	7 (44%)	8 (67%)	
Promotes the welfare and development of junior faculty			0.869
0	5 (31%)	4 (33%)	
1	11 (69%)	8 (67%)	
Refusal to violate one’s personal and professional code of conduct			0.539
0	7 (44%)	3 (25%)	
1	9 (56%)	9 (75%)	
Appreciates and respects the diverse nature of research subjects and/or patients, and honors these differences in one’s work with them			0.309
0	8 (50%)	4 (33%)	
1	8 (50%)	8 (67%)	
Attends faculty meetings, seminars, and student research presentations as a reflection of support			0.067
0	11 (69%)	4 (33%)	
1	5 (31%)	8 (67%)	
Works collaboratively and respectfully within a team to the benefit of improved patient care or to the contribution of research			0.408
0	7 (44%)	3 (25%)	
1	9 (56%)	9 (75%)	
Participates in corrective action processes toward those who fail to meet professional standards of conduct			0.031
0	10 (63%)	3 (25%)	
1	6 (38%)	9 (75%)	
Does not seek to advance one’s career at the expense of another’s career			0.021
0	8 (50%)	2 (17%)	
1	8 (50%)	10 (83%)	
Volunteers one’s skills and expertise for the welfare of the community			0.149
0	9 (56%)	3 (25%)	
1	7 (44%)	9 (75%)	
Meets commitments and obligations in a conscientious manner			0.011
0	9 (56%)	3 (25%)	
1	7 (44%)	9 (75%)	
Respects the rights, individuality, and diversity of thought of colleagues and students			0.026
0	7 (44%)	2 (17%)	
1	9 (56%)	10 (83%)	
Meaningfully contributes to the teaching mission of the department and the college of medicine			0.028
0	9 (56%)	2 (17%)	
1	7 (44%)	10 (83%)	
Shows compassion			0.046
0	6 (38%)	1 (9%)	
1	10 (63%)	10 (91%)	
Demonstrates adaptability in responding to changing needs and priorities			0.313
0	6 (38%)	2 (18%)	
1	10 (63%)	9 (82%)	
Promotes justice in the healthcare delivery system by demonstrating efforts to eliminate discrimination in healthcare			0.091
0	7 (44%)	2 (18%)	
1	9 (56%)	9 (82%)	
Respects patient autonomy and helps them make informed decisions			0.313
0	6 (38%)	2 (18%)	
1	10 (63%)	9 (82%)	
Assumes leadership in patient management			0.097
0	8 (50%)	2 (18%)	
1	8 (50%)	9 (82%)	
Recognizes one’s own limitations			0.097
0	8 (50%)	2 (18%)	
1	8 (50%)	9 (82%)	
Assumes personal responsibility for decisions regarding patient care			0.421
0	6 (38%)	2 (18%)	
1	10 (63%)	9 (82%)	
Participates in activities aimed at attaining excellence in patient care			0.560
0	6 (38%)	3 (27%)	
1	10 (63%)	8 (73%)	
Reports medical or research errors			0.071
0	8 (50%)	2 (18%)	
1	8 (50%)	9 (82%)	
Acts in ways that show a commitment to confidentiality			0.732
0	4 (25%)	2 (18%)	
1	12 (75%)	9 (82%)	
Adopts uniform and equitable standards for patient care			0.046
0	6 (38%)	1 (9%)	
1	10 (63%)	10 (91%)	
Demonstrates empathy			0.386
0	5 (31%)	2 (18%)	
1	11 (69%)	9 (82%)	
Advocates a patient’s or research subject’s interest over one’s own interest			0.586
0	8 (50%)	4 (36%)	
1	8 (50%)	7 (64%)	
Discloses conflicts of interest in the course of professional duties and activities			0.261
0	7 (44%)	3 (27%)	
1	9 (56%)	8 (73%)	
Is professionally attired in a manner that is respectful of others			0.589
0	7 (44%)	4 (36%)	
1	9 (56%)	7 (64%)	
Responds to constructive criticism by working to improve one’s capability in the area criticized			0.261
0	7 (44%)	3 (27%)	
1	9 (56%)	8 (73%)	
Commits to implement cost-effective patient care			0.103
0	8 (50%)	3 (27%)	
1	8 (50%)	8 (73%)	
Represents information and actions in a truthful way			0.535
0	6 (38%)	3 (27%)	
1	10 (63%)	8 (73%)	

## References

[1] Gilson A, Safranek CW, Huang T, Socrates V, Chi L, Taylor RA, Chartash D (2023). How does ChatGPT perform on the United States Medical Licensing Examination (USMLE)? The implications of large language models for medical education and knowledge assessment. JMIR Med Educ.

[2] Wong K, Fayngersh A, Traba C, Cennimo D, Kothari N, Chen S (2024). Using ChatGPT in the development of clinical reasoning cases: a qualitative study. Cureus.

[3] Kıyak YS, Emekli E (2024). ChatGPT prompts for generating multiple-choice questions in medical education and evidence on their validity: a literature review. Postgrad Med J.

[4] Veras M, Dyer JO, Rooney M, Barros Silva PG, Rutherford D, Kairy D (2023). Usability and efficacy of artificial intelligence chatbots (ChatGPT) for health sciences students: protocol for a crossover randomized controlled trial. JMIR Res Protoc.

[5] Khan RA, Jawaid M, Khan AR, Sajjad M (2023). ChatGPT - reshaping medical education and clinical management. Pak J Med Sci.

[6] Eysenbach G (2023). The role of ChatGPT, generative language models, and artificial intelligence in medical education: a conversation with ChatGPT and a call for papers. JMIR Med Educ.

[7] Dave T, Athaluri SA, Singh S (2023). ChatGPT in medicine: an overview of its applications, advantages, limitations, future prospects, and ethical considerations. Front Artif Intell.

[8] Liu J, Wang C, Liu S (2023). Utility of ChatGPT in clinical practice. J Med Internet Res.

[9] (2025). ACGME common program requirements (residency). https://www.acgme.org/globalassets/pfassets/programrequirements/cprresidency_2023.pdf.

[10] Project of the ABIM Foundation, ACP-ASIM Foundation, and European Federation of Internal Medicine (2002). Medical professionalism in the new millennium: a physician charter. Ann Intern Med.

[11] Blackall GF, Melnick SA, Shoop GH (2007). Professionalism in medical education: the development and validation of a survey instrument to assess attitudes toward professionalism. Med Teach.

[12] Akhund S, Shaikh ZA, Ali SA (2014). Attitudes of Pakistani and Pakistani heritage medical students regarding professionalism at a medical college in Karachi, Pakistan. BMC Res Notes.

[13] Pearson WG Jr, Hoagland TM (2010). Measuring change in professionalism attitudes during the gross anatomy course. Anat Sci Educ.

[14] Scherr R, Halaseh FF, Spina A, Andalib S, Rivera R (2023). ChatGPT interactive medical simulations for early clinical education: case study. JMIR Med Educ.

[15] Skryd A, Lawrence K (2024). ChatGPT as a tool for medical education and clinical decision-making on the wards: case study. JMIR Form Res.

[16] Yanagita Y, Yokokawa D, Fukuzawa F, Uchida S, Uehara T, Ikusaka M (2024). Expert assessment of ChatGPT's ability to generate illness scripts: an evaluative study. BMC Med Educ.

[17] Almazyad M, Aljofan F, Abouammoh NA (2023). Enhancing expert panel discussions in pediatric palliative care: innovative scenario development and summarization with ChatGPT-4. Cureus.

[18] Alkaissi H, McFarlane SI (2023). Artificial hallucinations in ChatGPT: implications in scientific writing. Cureus.

[19] Athaluri SA, Manthena SV, Kesapragada VS, Yarlagadda V, Dave T, Duddumpudi RT (2023). Exploring the boundaries of reality: investigating the phenomenon of artificial intelligence hallucination in scientific writing through ChatGPT references. Cureus.

[20] Falletta SV (1998). Evaluating Training Programs: The Four Levels: Donald L. Kirkpatrick, Berrett-Koehler Publishers, San Francisco, CA, 1996, 229 pp. Am J Eval.

